# How Are Sports-Trackers Used by Runners? Running-Related Data, Personal Goals, and Self-Tracking in Running

**DOI:** 10.3390/s21113687

**Published:** 2021-05-26

**Authors:** Armağan Karahanoğlu, Rúben Gouveia, Jasper Reenalda, Geke Ludden

**Affiliations:** 1 Faculty of Engineering Technology, University of Twente, 7522 NB Enschede, The Netherlands; r.h.gouveia@utwente.nl (R.G.); g.d.s.ludden@utwente.nl (G.L.); 2 Faculty of Electrical Engineering Mathematics and Computer Science, University of Twente, 7522 NB Enschede, The Netherlands; j.reenalda@utwente.nl; 3 Roessingh Research and Development, 7522 AH Enschede, The Netherlands

**Keywords:** sports tracking, running, goal achievement, running-related data, qualitative studies

## Abstract

The purpose of this research is to explore the roles that sports trackers and running-related data play in runners’ personal goal achievement. A two-week diary study and semi-structured interviews were conducted with 22 runners to explore how runners engage with their running-related data to set and achieve their running goals. We found that participants pursued and transitioned between different running goals as their needs, abilities, and surrounding environment changed. We also found multiple motivations that shaped the use of sports trackers. We identified two main categories in runners’ motivations for using trackers and data to achieve their goals. These categories were (i) documenting and tracking in running, and (ii) supporting goal-oriented reflections and actions, with various reasons for use while preparing for and during running. This study provides insights into the psychological effects of running-related data and signals practical implications for runners and developers of tracking technology.

## 1. Introduction

Using wearable technologies to monitor and track running behavior is currently common practice for many professional as well as recreational runners. Wearable sports trackers are capable of tracking different types of sports-related data (e.g., pace, cadence) compared to activity trackers (which provide feedback to people on number of steps taken) (e.g., [1]) and do not use additional external sensors to track users’ sports data. Janssen et al. [2] showed that over 85% of recreational runners use at least one mobile app or a sports watch to plan and complete runs. Meanwhile, a growing body of work explores the fundamental roles of technology in running practices [3,4,5]. Results show that runners are eager to learn about their running behavior by using the technology and the data the technology generates. Researchers have also been keen on generating and understanding the data, mostly by monitoring the running activity itself by measuring various biomechanical (e.g., joint angles) and physiological (e.g., heart rate) data. These developments translate running practices into aspects that are easy to track in “measurable units” [6].

The available data, in a broad sense, makes sports technology a helpful and effective companion in improving performance and physical condition [7], enhancing motivation [8] and preventing injuries [9,10]. There has been a recent focus on measuring and improving running technique through technology, as technique is an essential determinant of running performance and running-related injuries [3]. Research shows that irrespective of how experienced a runner is, they need meaningful data, feedback, and guidance in a personalized way that allows making relevant interpretations about performance and personal goals [1].

Self-driven goals have a positive effect on the motivation of recreational runners. Roberts et al. [11] found that goals and the meanings individuals attach to achieving their goals play a significant role in determining the amount of time, effort, and personal resources individuals spend on goal achievement. For instance, setting weekly-distance goals and receiving performance feedback increased recreational runners’ weekly running distance [12]. Goals can be very personal (e.g., running a personal best time in 5 km) and require intrapersonal goal adaptation (e.g., running 5 km between 20–25 min) [13]. Duda [14] portrays that setting a performance goal (e.g., performing a 5 km run within a set time) does not always motivate people towards achieving a goal. Merely competing with oneself and achieving/improving personal goals [15] can also be the motivation for running. For instance, people can be motivated by a process (e.g., working towards running 5 km) or a competitive outcome (e.g., being in the top 100 in a 5 km race). Moreover, most runners perceive running as an enjoyable activity [16], a good way of escaping from stress [17], and as an activity that contributes to personal health and quality of life [18].

For many runners, running-related data provide an easy way to observe progress and reflect on self-driven goals [19]. Sports-tracking technology can provide its users with different metrics to reflect on goals, which is why researchers investigate the role of interactive technology in running-tracking. Whooley et al. [20] highlight the potential for self-tracking devices to support reflection in action, where people check their data and change their behaviors while tracking.

However, reflecting on goals is not enough for goal achievement. It involves an interplay between personal (e.g., skills) and goal-related (e.g., goal orientation) variables [21]. Therefore, setting too difficult and non-achievable training goals (e.g., constantly increasing weekly running volume) increases the risk of failure [22]. Such a failure can result in detrimental emotional effects that jeopardize the health benefits of being active [23]. Niess and Wozniak [24] highlight the importance of supporting people’s transitions between goals, proposing that they can contribute to long-term interests in self-tracking practices. Therefore, people should be supported in articulating and eliciting changes in their goals and planning how trackers can best support them in meeting these goals [25].

So far, the opportunities for these and other functions of technology and running-related data to support runners in self-setting and achieving goals, have been underexplored. Research on tracking technology has predominantly focused on the technical development of sports-trackers rather than understanding how data tracked could help runners achieve their running-related goals. We think this is worth studying because knowing one’s progress is not always sufficient to set or reflect on running-related goals. While sport psychology consultants can help the runners to use wearable technology properly [26], the data availability does not always turn the knowledge into action [27]. In some cases, more data might even be regarded as a burden and a way of self-surveillance in running activities [28].

Hence, in this paper, we aim to understand how runners use their sports trackers to help them set and achieve their running goals. In the following sections, we explain the method and results of the participant study in which we investigated the effects of running-related data on setting and achieving running-related goals.

## 2. Materials and Methods

### 2.1. Participants

We recruited participants through our personal networks, online networks for running (e.g., Strava) and from a local participant pool (e.g., local running clubs). To qualify, participants had to use some sort of wearable technology to track their runs and commit to completing at least two weekly runs over the duration of our study. Our target sample size for the study was 20 participants, which is a common target size in research that uses diary studies for understanding the roles and uses of technology [29,30].

A total of 32 people volunteered to participate in the study. We communicated the details of the study by sending out personal emails to the volunteers. Of these, eight did not qualify for the study (4 did not use technology to track their runs, four had not been running recently), and a further two dropped out of the study. Eventually, 22 participants (Table 1) completed the full study (10 female, 12 male, median age = 38, min = 24, max = 58). Participants had all been running for at least one year (median = 13 years of running experience, maximum = 26 years) and using their current sports-tracker for at least four months (median = 2 years, max = 5 years, see Table 1 for the tracking devices and applications used by participants). By the time of this study, all the participants were living in the Netherlands.

### 2.2. Data Collection Procedure

We selected two methods to inquire into runners’ experiences with running-related data in goal achievement: interviews and diaries. This decision is in line with the suggestions of McGannon et al. [31], who recommend coupling interviews with an “additional range of knowing” in sports and exercise qualitative studies. Self-report diary-logs have been considered a useful method to understand physical activity [32] and sports [33,34] practices. Diaries have been used extensively in research to sample people’s behaviors and intent in situ to support reflection on and recall events [35]. They have been found to augment people’s abilities to recall more events [36], describe events in detail and make associations between events [29].

The proposed approach offers at least two benefits for understanding tracker use: it (1) minimizes the risk of missing or overlooking important moments and (2) enhances runners’ ability to recall detailed information about their experiences with trackers. Ethical approval was obtained from the local ethical committee before contacting participants.

**Figure 1:**
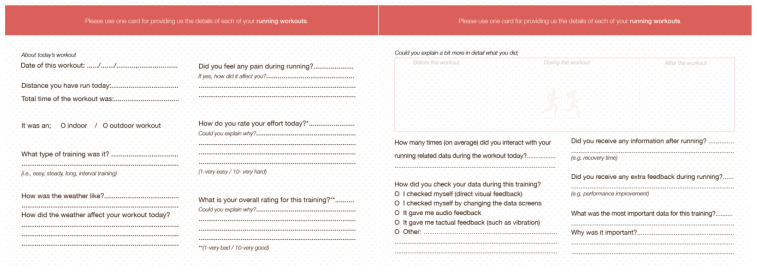
Layout and questions of the running-related data experience study (diary).

Participants were given a diary for two weeks and asked to fill in information about each of the runs they took within this timeframe. This included: (a) general information about a run, such as the distance, data and type of run (e.g., interval training, easy run), and (b) how trackers were used during and following runs (see Figure 1 for a layout of the diary). Participants were asked to complete this information shortly after completing a run. While similar studies have given diaries/diary exercises to participants for one week (e.g., [34]), we opted for an additional week in an attempt to log at least four of our participants’ runs, assuming that recreational runners complete, on average, 3.7 ± 1.6 runs per week [37].

Two semi-structured interviews were conducted with each participant: one before starting the study and another immediately following its completion. In the first interview, participants were introduced to the study and their written consent to participate in the study was collected. During the interviews, we asked the participants about their experience in running and running routines. In the second interview, participants were asked to choose at least two runs from the diary and describe: (a) some details for each run (Figure 1-left image) and (b) how trackers and running data were used for planning and completing runs (Figure 1-right image). During the interviews, the diary was used as a prompt to help participants recall details of runs. We encouraged the participants to open their running apps and use data from the run to further support their recollections. Apart from these, we did not ask the participants to provide us with their running-related data.

The first and second author conducted the interviews. Interviews were audio-recorded, and all data collected from participants were anonymized. The average duration of audio recordings for the first interview was 20 min (min = 12, max = 42 min), and the second interviews 33.2 min (min = 22, max = 52 min). Participants received a €20 gift card for completing the study.

### 2.3. Data Analysis

The analysis of interviews followed a thematic analysis [38]. Interviews were transcribed and stored in separate Excel sheets. The first round of interviews was inductively coded to arrive at participants’ experience of running and goals for running. The goals of runners were analyzed to determine sub-themes. Considering the similarities between the goals, we then grouped these units into main themes (e.g., achieving a specific performance goal).

The second interview and the diary of each participant were analyzed simultaneously. This analysis was done concurrently by the first and second author to ensure the reliability and validity of the results [39]. First, the transcriptions were read line by line and were coupled with the diary data of each participant. Each participant logged between 2–7 runs per week in their diary, giving us multiple points of analysis for the roles that trackers had in people’s lives. Raw data units (e.g., quotations, texts participants wrote in the diaries) in self-reports and diaries were thematically analyzed (e.g., checking if one’s effort is appropriately tracked), merged into the main themes (e.g., documenting and tracking runs) and were categorized under preparing for and during running training episodes. In cases of misalignment and disagreement, the first two authors made an additional round of iterations and discussion. The prevalence of each theme was measured by counting the number of times in which each theme appeared in participants’ interviews.

## 3. Results

We organize our findings based on our interview structure. We begin with the results from the first round of interviews, where we describe participants’ motivations and goals for running and how these goals shape trackers use. Next, we focus on the results from the second round of interviews, where we describe how trackers and data were used to support the planning and completion of runs.

### 3.1. Running Motivation

In line with previous studies [1], we measured participants’ motivation towards undertaking sport with the Sports Motivation Scale [40] to understand how motivated the participants are for performing in running. These results were used to characterize the subject population. As described in [40], the scale measures people’s motivation towards the sports activity according to three levels: intrinsic motivation (e.g., engagement in running for self-satisfaction), extrinsic motivation (e.g., engagement running for the praise from others), and amotivation (e.g., non-engagement due to lack of good reasons to continue running). Results showed that the participants of our study had high confidence in their capabilities of running. They were more intrinsically motivated to run (mean *M* = 4.64 out of 7.00, standard deviation *SD* = 0.74) than extrinsically motivated (*M* = 2.95 out of 7.00, *SD* = 0.85), meaning that running was inherently satisfying and enjoyable for them.

### 3.2. Goals for Running

We classified participants’ goals for running into two main categories: (1) achieving a particular performance goal and (2) keeping running as a habit. In Table 2, we break down these categories further, include examples for each of the categories and their frequency of occurrence.

#### 3.2.1. Achieving a Specific Performance Goal 

More than half of our participants (*n* = 16) were interested in reaching individual achievements. Examples included completing a particular running event (e.g., a marathon) or improving one’s running performances. Often, performance goals were specific and quantitative, and easily tracked by trackers (e.g., reducing one’s current 10 km running time by 30 s per kilometer or increasing one’s weekly running distance from 50 km to 70 km). Participants with performance goals were also usually using trackers alongside training programs. In most cases, these programs were planned with a trainer. Only in one case was the program actually created by the tracker.

#### 3.2.2. Keeping Running as a Habit 

Participants (*n* = 12) also frequently described being interested in maintaining running as a habitual activity. More than wanting to achieve personal records or performance goals, the goal here was to keep running as a consistent activity in one’s daily life.

Sometimes these goals were part of long-term, even lifelong preventive health efforts, such as exercising to keep one’s blood pressure under control (P11) or stress levels reduced (P4). This highlighted the centrality that running had in the routines of some participants. For example, P5 and P10 described their runs as an essential part of their weekly routines and creating specific strategies towards how running could be included in an upcoming day or week. Examples included planning to run at least once during weekends (P4), running every odd day after leaving work and bringing sports equipment to one’s workplace to facilitate running (P17). One interesting case is P14, who described running at 7 a.m. every Saturday for the past couple of years, regardless of weather conditions.

Maintaining running as a habit was not always an easy task, and sports-trackers were often sought to support runners in sticking to their plans. For instance, P4 described using his tracker to seek inspiration for running. For others, collecting data was a way of checking if they had a constant performance in runs and ultimately keeping themselves accountable for this. 

#### 3.2.3. Interweaving between Goals 

Interestingly, most participants (*n* = 19) described having multiple, concurrent running goals. For example, P15 described her runs as a mix of performance-oriented goals (e.g., running 5 km under 25 min) and casual runs, where the goal was to meet up and run with friends or simply have fun:

‘*My runs are mixed. Some of them are for the training plan, but others are just kind of for me. It is like a different training where you are just running for yourself and can have your style and do what you want (…) it keeps things fun, and it kind of motivates me to mix things*.’

(P15)

Participants often transitioned between different goals as their needs, running abilities, and surrounding environment changed. Examples included initially wanting to reach a new record running distance, then switching to a casual run, where the goal was simply to have fun due to weather conditions. Transitions were also the result of life-changing events, such as motherhood or injuries, where previously pursued goals no longer felt attainable:

‘*When I only had one child, I would hate it when I could not go for the run. I would be a bit stressed about it (…), but after having another two (children), running has a different purpose. It’s more leisure. It’s more like, forgetting about your day at work and being outside. I’m a different runner now, one that doesn’t go after records but looks for fun*.’

(P15)

These transitions shaped how trackers and tracked data were used. Four participants described switching back and forward between different tools that better met their different goals needs, such as switching from an activity tracker, ‘*because it just tracks the steps at the end of the day*’ to a sports watch that ‘*helped to better see how my running is going and track it*’ (P7). Switching between tools was often motivated by features and metrics better aligned with one’s ongoing goal (e.g., ‘*The running app has these virtual coaches that help me plan my runs and the sports watch has better interval planning*’, P15) and perceived accuracy (e.g., ‘*The sports watch has better sensors for tracking my heart rate*’, P17).

Switching between tools was also an attempt to keep one’s running data separated by goals. For example, P17 would use her sports watch to track runs related to her running plan and her activity tracker to track runs that were not part of this plan, such as slower runs with friends and family members. Even participants who only used one tracking device were found to separate their data by goals. As further described by P17, this separation created a clear conception of the self-being tracked: ‘*The sports watch data shows my serious runner side and the activity tracker is about the fun side... the slower runs with friends and family*’. Keeping this separation was important to reflect on one’s performance, but also to express one’s identity to others:

‘*I share my serious runs with my Strava friends, but not the ones from the activity tracker (…) I don’t want them to see that side of me*.’

(P17)

### 3.3. Usage of Running Trackers to Support Runs

Next, we looked at how sports trackers, and respective data, were used to support participants runs. We classified the use of trackers and data into two main categories: (1) documenting and tracking runs and (2) supporting goal-oriented reflections and actions.

#### 3.3.1. Documenting and Tracking Runs 

Nearly all participants (*n* = 21) were found to use their trackers for documenting purposes. In this case, participants were mostly interested in documenting their running activities rather than reflecting or trying to learn something from their data. Documenting, for some (*n* = 6), was an attempt to continue tracking one’s runs, with the hope that trackers would offer better support to reflect on that data somewhere in the future. Some participants (*n* = 4) wanted to share their efforts with family and friends and simply have them tracked for future reflection and learning. We looked at how these uses unrolled while preparing for and during runs (Table 3).

##### Documenting Practices when Preparing for Running

All participants checked if their trackers were set up adequately to track an upcoming run. This involved checking if the sensors of the specific watch were on (e.g., Global Positioning System—GPS), and battery levels would allow tracking the full duration of a run. The inability to adequately track a run was seen as a missed opportunity. Some participants felt this way because they were aggrieved by the amount of physical activity they were performing and somehow wanted to underline this effort (*n* = 6). As described by P6, forgetting to track one’s run felt like the effort was not accounted for:

‘*This is the most important thing to do when preparing for runs. It feels like the effort is lost if it is not tracked or not tracked accurately*.’

(P6)

Depending on the goal of a run, participants chose specific tools and data (*n* = 5) for supporting their goals (as mentioned in Section 3.2.3). These decisions were normally made shortly before a run and involved thinking about which data would be useful to answer a specific question or attend to a particular goal. Choosing appropriate data entails that one might need to avoid tracking specific data. For example, heart rate might not be useful (and even be detrimental) for a particular “fun run”.

##### Documenting Practices while Completing a Run

We found participants use, but sometimes avoid, using their trackers in specific ways when documenting their runs (*n* = 12). It is important to recall that people’s goals here were not necessarily to adjust or monitor their behaviors, but rather to simply track their runs. When this was the case, interacting with a tracker while running was often avoided. Tracking was often described as an activity competing for one’s attention with an ongoing run. It was described as distracting (e.g., P5) and confrontational (e.g., P17). The feedback given to participants pushed them towards comparisons and adjustments in behaviors, which was not their intention when simply wanting to document a run. Rather, when participants did check their trackers, they did so to confirm that their runs were being tracked (i.e., checking if GPS connection is still working or watch is still tracking). For instance, P15 mentioned being annoyed about the audio feedback that her tracker gives her, indicating high heart rate is high. Her coping strategy was simply to ignore the feedback. Another example was given by P15, who described the distractions that tracking could cause:

‘*I don’t check it (smartwatch) that often while running. It’s like they say... watching a pot that never boils. Checking makes me think of what I still have to do, instead of focusing on what I’m doing now*.’

(P15)

When participants were tracking for documentation, tracked data were less frequently checked, and there was room for surprising insights. This contrasted to experiences within stricter, performance-related runs, where trackers were often persistent reminders of what one should be doing. As mentioned by one participant, the fun of tracking without a goal was learning something unexpected or something that was not in the back of one’s mind:

‘*On Thursday, I do what my trainer tells me. But on Sundays and Tuesdays, I do my own thing. Sometimes, I do some uphill and downhill training. Or just run for fun. (…) I don’t have any goal in mind, and I don’t check my watch that often. Maybe just at the end to see how I did, and it’s like, “oh nice, I did this”, but it’s not something I am always checking to see if I am on track. Even without a strict goal, it helps me understand myself better. It can show me if I am doing well compared to normal or if I am running slower*.’

(P12)

Some participants (*n* = 8) seemed to be using trackers this way because they were aggrieved with their past experiences with tracking. P4, for example, avoided checking his tracker when feeling that his heart rate was high, as it would confirm his concerns during a run which he does not want to interrupt:

‘*In these circumstances, you just want to get this done. I think I did not slow down so much, but because I was already a bit nervous or scared that route, it would demoralize me while running*.’

(P4)

#### 3.3.2. Supporting Goal-Oriented Reflections and Actions

All participants used their trackers to reflect on their running goals and to help them take action towards these goals when preparing for and during running (see Table 4). In the following section, we describe the ways in which trackers were used for supporting these reflections and actions.

##### Goal-Oriented Reflections and Actions When Preparing for a Run

Participants highlighted the importance of reflecting on past data while planning an upcoming run (*n* = 16). Reflections were often directed towards one’s personal running goals, such as reviewing one’s past runs to see how one was progressing towards a certain running pace (e.g., P10) or distance goal (e.g., P15). These participants followed a training plan and wanted to see if they were following the goals set out by that program. Interestingly, reflections were often described as focused on the short-term, happening immediately or shortly after data were gathered from a run. As emphasized by P13, running was impacted by multiple factors that sports-trackers did not easily track (e.g., what the weather was like or how one’s feelings impacted performance). Leaving reflection for a later time can make one overlook or forget important insights.

Participants described setting up their trackers in particular ways to keep them on track with their running goals while running. Trackers, in these cases, were used to help participants identify opportune moments for action when running (*n* = 9), such as when one should start running slower to avoid ‘burning out’ (e.g., P7) or faster to keep up with a target pace (e.g., P10). Given the difficulty of identifying these moments, some participants mentioned using their trackers to alert them towards potential action moments. Examples included having a watch that gives an alert once a heart rate was above or below a target zone or when one was out of a target running pace. Alerts helped participants identify moments of action and avoided frequent engagements with their tracker to see if one was on track.

Participants also indicated that they use their trackers to forecast their performance in an upcoming run (*n* = 5). For instance, P8 checked her resting heart rate before running 20 km to estimate how fast she would be able to run. Predictions were strongly connected to participants’ perceptions of their abilities. Trackers helped them shape the perception of their abilities. For instance, P12 knew he could run a certain distance within a specific timeframe after seeing himself achieve that in a tracked run. Others described looking back and combining multiple tracked parameters, such as resting heart rate, weather and sleep patterns, to learn how these might have influenced performance. As described by P1, this helped to adjust their training practices (using strategies such as avoiding running, or changing a running plan following a stressful day at work, as reflected by a relatively high heart rate).

Participants described being inspired and motivated by other people’s runs on social networks (*n* = 5). For some, this was due to shared goals. P15, for example, shared a weekly running goal with friends. Seeing his friends complete their weekly goals motivated him to run. Others described being inspired by (and even copying) other people’s running paths or paces. As described by P18, seeing new running routes was a motivation to try something new and get out running. Interestingly, social networks were not only used to motivate running but also to justify deviations from goals:

‘*None of my friends had any runs this week. That made me felt less guilty for not reaching my 20 km this week*.’

(P7)

##### Goal-Oriented Reflections and Actions during a Run

While running, goal-oriented tracking was mostly tailored towards self-monitoring and reflection-in-action. The majority of participants (*n* = 21) mentioned that a primary motivation for using a sports-tracker was to check how they were doing and see if adjustments needed to be made while runs took place (*n* = 14). This involved checking data to see if behaviors matched certain goals and adjusting, for example, running pace accordingly. Most trackers did not have specific suggestions on what adjustments should be made, making participants check their watches frequently to see if action needed to be taken.

Self-monitoring was commonly described as impulsive, frequent, and something done unconsciously (*n* = 16). For example, P7 described checking her tracker frequently, over 20 times during 30-min runs. Some runners seemed to be using trackers this way to seek reassurance during moments of uncertainty, such as to see if one’s heart rate was within a target zones while running at a faster pace than usual (e.g., P12), or if one’s pace was not considerably lower while running during a rainy night (e.g., P4). Frequent checking would allow the runner to identify discrepancies during running and adjust their behavior accordingly. Others seemed to check their trackers frequently when reaching or anticipating moments of accomplishment and success. Participants seemed to seek acknowledgement of their achievements through their trackers, as described by P2:

‘*When you think you’re really at a good pace, and then see if that’s true [when checking the tracker] it feels good to see that*.’

(P2)

Interestingly, monitoring runs was not always seen as a positive practice (*n* = 8). Some described the frequent checking of one’s tracker as an obsessive practice, often taking away from one’s judgment. P22, for example, described being too focused on his data while running: ‘*I was focusing too much on the data, and that was affecting the result*.’ P18’s running coach suggested him stop running with a tracker for two months to re-gain awareness of his capabilities:

‘*I was putting too much effort into thinking about the data and thinking about hitting the numbers and sometimes forgot about the run, so I stopped running with it for two months*.’

(P18)

In such cases, trackers were sought by people for assistance or to provide answers but rather opened space for more questions and doubts, distracting from runs. This led some participants to avoid checking trackers altogether while running:

‘*I feel horrible when seeing that I am not keeping up with a pace that I planned (...) so I start thinking if I should push myself harder on the next kilometer or punish myself by running slow (...) So you just avoid looking at it at all and check once you’re done because it influences me in the wrong way (…). It’s just not that helpful, is it?*’

(P13)

The lack of specific suggestions also made it difficult for participants to understand the information they saw on their watches (e.g., what does a high heart rate say about my chances of completing a 50 km run?). Runners dealt with this in different ways. Some drew normative comparisons with their data, such as comparing one’s heart rate with that of runners with similar running experience (P18) and friends running with them (P4). Having a normative comparison helps a runner understand if they are doing well or not. Others took into consideration different everyday factors when weighing the need to take action and deciding what action to take. This was, for example, the case for P19, who described reflecting on her workday to decide if she should make adjustments to her pace, following a high heart rate: ‘*I gave myself a minute to go a little bit faster. I had a good day and had rested well, so I went with it*.’

## 4. Discussion

This study aimed to understand how runners use sports-tracking devices to help them plan and achieve their running goals, and how data collected by these devices are embedded in their practices. Our study started by investigating participants’ different running goals. We found participants to run for two main reasons: achieve specific performance goals and keep running as a habit. These results are not necessarily surprising. Previous research has highlighted the importance of performance goals in motivation (e.g., [14]). In most cases, achieving personal goals, such as improving running pace or distance, is what motivates runners to keep running [15]. Furthermore, merely enjoying runs [16] and improving one’s overall health [18] have also been found as important reasons to motivate running.

Perhaps more surprisingly, participants expressed pursuing multiple goals simultaneously and transitioning between different goals as their needs, abilities and surrounding environment changed. Runners would often change between data-intensive performance goals and less-burdensome documentations of their runs as their motivation for running changed. Sports tracking devices generally failed to fully support these goal changes, forcing some runners to switch to different tracking tools that better matched their different goals. These insights resemble those of previous work, which has found people transition naturally between different tools as their goals change [24].

However, more commonly, we found that runners used one sports-tracker and found workarounds to support their goals and new ways for using their trackers as their goals changed, even if not optimally supported by their tracker. We advocate that the design of sports-trackers should include highlighting the extent to which they are able to support particular running goals. This would help runners decide how useful a particular tracker could be for addressing their goal before they begin tracking. These insights also seem to support the importance of flexible tracking approaches, where runners can experiment with the ways their trackers collect and display data towards supporting different short- (e.g., particular training) and long-term (e.g., training for a marathon) goals. One could imagine trackers having different configurations for these different goal types.

Our study also highlighted a number of ways in which trackers were used to help runners reach their different running-related goals. Trackers were used predominantly for documenting purposes (as in [41]). This involved using a tracker to simply track one’s runs, rather than for reaching or reflecting on one’s goals as running happened. Tracker use was also driven by micro-reflections, where data were briefly checked following a run or while planning for an upcoming run. Interestingly, these reflections were avoided while runs were happening. These findings contrast with recent models of self-tracking (e.g., [42]), which have found that people collect data, reflect and take action simultaneously, as their behaviors unroll. Instead, runners seem to create a stricter separation between these different actions, first collecting data and then reviewing and planning future courses of action after runs were completed. Trackers seemed to be used this way because of the difficulties participants have in interpreting and making data actionable. This did not mean that trackers did not help runners reach or keep in line with their goals while running. Rather, trackers were used strategically while running. Several participants set up their trackers to notify them if they were deviating from their goals and to identify opportunities for action (e.g., by receiving alerts).

Based on our insights, we derived three practical implications for developers of coaching and sports-tracking systems:

*Support goal-changes*: We found that nearly all participants changed their running-related goals over time. These changes were triggered by changes in motivation (e.g., changing from performance outcome to learning goals as explained in [43]) and changes in running abilities (e.g., going through injuries or get pregnant). We believe that fully supporting goal-driven tracking requires supporting goal changes and evolutions that happen through many changes and transitions that constitute human life. Runners should be supported in articulating and eliciting changes in their goals and be offered actionable guidance, specifically towards meeting these goals. Trackers could, for example, use people’s data and deep personalization modelling (as suggested in [44]) to learn users’ patterns and trigger them to reflect and set new goals when identifying changes in their patterns.

*Supporting multiple ongoing goals*: We also found nearly all participants pursue multiple, different goals at once. We propose that running-related data should be presented such that it connects with the runners’ current needs, goals and perceptions of their abilities. A tracker that knows people’s goals could adjust its behavior to provide feedback in terms of factors that people care about, even if these things might not be directly about improving performance. One idea could be to maintain user profiles that include information about goals and aspirations that people have, as well as how trackers could be used to help them in reaching those goals. Runners could be encouraged to choose a device or support ‘system’ (such as trackers, apps or friends) that would best support them in their running-related goals.

*Support just-in-time adjustments*: Our study highlighted the dual nature of feedback: while helping runners in planning their runs and identifying discrepancies in their goal pursuits, checking a tracker was often avoided when running took place. Data were seen as distracting, confrontational and detrimental towards the completion of one’s runs. Feedback was often given at moments that were considered inappropriate, and data often misaligned with participants’ goals. We advocate further exploration on the timeliness of data. We suggest that the details of behavioral support (e.g., content and timing of feedback) could be tailored based on the collected data and triggered by the system [45]. Trackers can make use of triggers only if the runner needs in-running feedback on a certain parameter. This way of triggering could prevent the runner from unnecessary and constant data-checking while running. These results seem to highlight the importance of having observable, actionable information that users do not have to spend much time reflecting on or making sense of during runs, as also suggested by Gouveia et al. [46].

## 5. Conclusions

In conclusion, our study investigated runners’ practices when using a tracking device and how they integrated the data provided by these devices in their running routines. As such, it looked at the running-related data from a different angle than traditional biomechanical or physiological studies. In most studies, informative and effective roles of running-related data are explored. In addition to these informative roles of data, we explored their psychological effects and how these effects influence the ways runners use their trackers. We believe that bringing these fields of study together can result in meaningful running-related data presentations at specific moments in time to comply with runners’ needs, wishes and goals, rather than a technology-pushed presentation of specific sets of data.

We propose that technology developers should be aware of the psychological effects of running-related data on runners. Future research could examine how sports technology facilitates ignorance of data while still informing the runner that some data are important to track and be aware of, especially when these data are of considerable importance because they relate to their goals. Our study complements related studies in our sample selection (e.g., [1,8]). However, it has a limitation in that we did not sample participants with specific levels of running experience. We recruited runners who used a wearable sports tracker to track their runs. This led to a sample with a wide variety in running experience (median = 13 years of running experience, maximum = 26 years). As such, the results only reflect the runners who use wearable sports trackers with a certain number of years of experience in recreational running. A follow-up study could look into the effects of experience levels on the use of tracker and running-related data and its psychological effects on running motivation.

## Figures and Tables

**Table 1:** Overview of participants.

Demographics			Running Experience and Current Trackers Use		
PN	Gender	Age	Years Running	Tracking Device	Apps Being Used
P1	Female	48	>7 years	Polar M 400	Strava
P2	Female	35	>3 years	TomTom watch	Strava and Runkeeper
P3	Male	38	>15 years	Garmin Fenix 3HR	Strava, RunAnalyze, TrainAsOne
P4	Male	28	>3 years	Garmin Forerunner	Strava and Nike Running
P5	Male	56	>25 years	Garmin (Not sure which model)	Not using any app
P6	Female	24	>1.5 years	Garmin (Not sure which model)	Strava and Runkeeper
P7	Female	28	>6 years	Polar smartwatch + Heart Rate band and Fitbit Versa	Polar and Strava
P8	Female	43	>13 years	Garmin Vivo Active (2 years)	Strava and Runkeeper
P9	Male	42	>19 years	Garmin (Not sure which model)	Not using any app
P10	Female	27	>7 years	TomTom sports and it’s app	Strava and Tomtom
P11	Male	49	>7 years	Garmin (Not sure which model)	Strava and Garmin
P12	Male	52	>5 years	Garmin (Not sure which model)	Strava
P13	Male	43	>20 years	Garmin (Not sure which model)	Not using any app
P14	Male	58	>26 years	Garmin Forerunner	Not using any app
P15	Female	24	>1 years	Garmin Forerunner	Garmin Connect
P16	Male	26	>10 years	Tomtom watch	Strava
P17	Female	43	>7 years	Garmin (Not sure which model)	Garmin Connect
P18	Male	23	>15 years	Garmin Forerunner 235	Strava
P19	Female	38	>20 years	Garmin (Not sure which model)	Not using any app
P20	Male	52	>24 years	Apple Watch	Nike running and Runkeeper
P21	Male	22	>9 years	Garmin Forerunner (235)	Strava and Garmin connect
P22	Male	28	>3 years	Garmin (945 XT)	Strava and Garmin connect
		37.59	>13 years		

**Table 2:** Goals for running.

Goals	Sub-Goals	Example (Having the Goal of)	# P
Achieving a specific performance goal	Improve running pace	Running 30 s/km faster than one’s current record for a particular distance (e.g., 10km)	8
	Complete a particular event	Completing a half marathon	8
	Improve a running distance	Running 5 km longer than one’s longest running distance	3
Keeping running as a habit	Maintain activity level	Running at least twice a week	10
	Improve overall health	Recover from running injury	4

**Table 3:** Use of tracker and data in documenting and tracking in running.

Moment	Practices with Trackers and Data	Example	# P
Preparing for running	Checking if one’s effort is appropriately tracked	Checking Global Positioning System (GPS) connection	6
	Choosing appropriate data and tools for supporting a specific goal	Switching between a sports watch to track “serious” runs and an activity tracker to track slower runs	5
During running	Using and avoiding interaction with trackers	Hiding one’s heart rate data to avoid distractions	12

**Table 4:** Use of trackers and data in goal-oriented reflections and actions about running.

Moment	Reasons for Use	Example	# P
Preparing for running	Planning through micro-reflections	Reflecting on past data while doing planning	16
	Facilitating action	Setting the device to give alerts to be within the goals of the run	9
	Forecasting one’s performance	Reflecting on past/recent data to forecast upcoming performance	5
	Drawing inspiration through social networks	Being inspired and motivated by other people’s runs on social networks	5
During running	Self-monitoring	Impulsive, frequent, or unconscious checking of data during running	16
	Reflection-in-action	Collecting data about the run while a run takes place.	14
	Supporting decisions	Checking data to make judgments about the performance and take actions accordingly	8

## Data Availability

The data presented in this study are available on request from the corresponding author. The data are not publicly available due to the GDPR agreements made with the participants.
